# A Comprehensive Study of Anomaly Detection Schemes in IoT Networks Using Machine Learning Algorithms

**DOI:** 10.3390/s21248320

**Published:** 2021-12-13

**Authors:** Abebe Diro, Naveen Chilamkurti, Van-Doan Nguyen, Will Heyne

**Affiliations:** 1 College of Business and Law, RMIT University, Melbourne 3001, Australia; abebe.diro3@rmit.edu.au; 2 Department of Computer Science and I.T., La Trobe University, Melbourne 3086, Australia; n.chilamkurti@latrobe.edu.au; 3 BAE Systems Australia, Adelaide 5000, Australia; will.heyne@baesystems.com

**Keywords:** cybersecurity, anomaly detection, the Internet of Things, machine learning, deep learning, blockchain

## Abstract

The Internet of Things (IoT) consists of a massive number of smart devices capable of data collection, storage, processing, and communication. The adoption of the IoT has brought about tremendous innovation opportunities in industries, homes, the environment, and businesses. However, the inherent vulnerabilities of the IoT have sparked concerns for wide adoption and applications. Unlike traditional information technology (I.T.) systems, the IoT environment is challenging to secure due to resource constraints, heterogeneity, and distributed nature of the smart devices. This makes it impossible to apply host-based prevention mechanisms such as anti-malware and anti-virus. These challenges and the nature of IoT applications call for a monitoring system such as anomaly detection both at device and network levels beyond the organisational boundary. This suggests an anomaly detection system is strongly positioned to secure IoT devices better than any other security mechanism. In this paper, we aim to provide an in-depth review of existing works in developing anomaly detection solutions using machine learning for protecting an IoT system. We also indicate that blockchain-based anomaly detection systems can collaboratively learn effective machine learning models to detect anomalies.

## 1. Introduction

The IoT consists of myriad smart devices capable of data collection, storage, processing, and communication. The adoption of the IoT has brought about tremendous innovation opportunities in industries, homes, the environment, and businesses, and it has enhanced the quality of life, productivity, and profitability. However, infrastructures, applications, and services associated with the IoT introduced several threats and vulnerabilities as emerging protocols and workflows exponentially increased attack surfaces [1]. For instance, the outbreak of the Mirai botnet exploited IoT vulnerabilities and crippled several websites and domain name systems [2].

It is challenging to secure IoT devices as they are heterogeneous, traditional security controls are not practical for these resource-constrained devices, and the distributed IoT networks fall out of the scope of perimeter security, and existing solutions such as the cloud suffer from centralisation and high delay. Another reason for this challenge is that IoT device vendors commonly overlook security requirements due to a rush-to-market mentality. Furthermore, the lack of security standards has added another dimension to the complexity of securing IoT devices. These challenges and the nature of IoT applications call for a monitoring system such as anomaly detection at device and network levels beyond the organisational boundary.

An anomaly is a pattern or sequence of patterns in IoT networks or data that significantly deviate from the normal behaviour. Anomalies can be contextual and collective points based on the sources of anomalies [3]. Point anomaly represents a specific data point that falls outside the norm, and it indicates random irregularity, extremum, or deviation with no meaning, often known as outliers. The contextual anomaly denotes a data point that deviates from the norm in a specific context such as in a time window. It means that the same normal observation in a given context can be abnormal in a different context. The contextual anomaly is driven by contextual features such as time and space and behavioural features such as the application domain. A collection of related data points, specifically in sequential, spatial, and graph data, that fall outside of normal behaviour forms collective anomalies. It is denoted as a group of interconnected, correlated, or sequential instances, where individuals of the group are not anomalous themselves; the collective sequence is anomalous. Anomalous events rarely occur; however, these events bring about dramatic negative impacts in businesses and governments using IoT applications [4].

As for protecting IoT and I.T. applications, intrusion detection systems (I.D.S.s) that alert abnormal events or suspicious activities that might lead to an attack have been developed. I.D.S.s can be divided into two main categories: anomaly-based and signature-based. With anomaly-based I.D.S.s, unidentified attacks or zero-day attacks can be detected as deviations from normal activities [5]. However, signature-based I.D.S cannot identify unknown attacks until the vendors release updated versions consisting of the new attack signatures [5]. This indicates that anomaly-based I.D.S.s are strongly positioned to secure IoT devices better than signature-based I.D.S.s. Moreover, there is a large amount of raw data generated by IoT devices, which leads to the process of identifying suspicious behaviour from data suffering from high computation cost due to included noise. Hence, lightweight distributed anomaly-based I.D.S.s play a significant role in thwarting cyber-attacks in the IoT network.

In recent years, using machine learning techniques to develop anomaly-based I.D.S.s to protect the IoT system has produced encouraging results as machine learning models are trained on normal and abnormal data and then used to detect anomalies [1,2]. However, building effective and efficient anomaly detection modules is a challenging task as machine learning has the following drawbacks:

- First, machine learning models, specifically with classical algorithms, are shallow to extract features that can truly represent underlying data to discriminate anomaly events from normal ones.
- Second, running machine learning models can consume extensive resources, making it challenging to deploy such models on resource-constrained devices.
- Third, it requires massive data for training machine learning models to archive high accuracy in anomaly detection. Therefore, machine learning models may not capture all of the cyber-attacks or suspicious events due to training data. This means that machine learning suffers from both false positives and false negatives in some circumstances.

However, with the advancement in hardware such as GPU and neural networks such as deep learning, machine learning has constantly improved. This makes it promising for anomaly detection emerging platforms such as blockchain.

This paper aims to provide an in-depth review of current works in developing anomaly detection solutions using machine learning to protect an IoT system, which can help researchers and developers design and implement new anomaly-based I.D.S.s. Our contributions are summarised as follows: first, we present the significance of anomaly detection in the IoT system (Section 2); then, we identify the challenges of applying anomaly detection to an IoT system (Section 3); after that, we describe the state-of-the-art machine learning techniques for detecting anomalies in the system (Section 4); finally, we analyse the use of machine learning techniques for IoT anomaly detection (Section 5). In particular, this paper also covers the federated learning technique that helps to collaboratively train effective machine learning models to detect anomalies (Section 4) and indicates that the use of blockchain for anomaly detection is a novel contribution as the inherent characteristics of a distributed ledger is an ideal solution to defeat adversarial learning systems (Section 5).

## 2. Significance of Anomaly Detection in the IoT

Over the years, anomaly-based I.D.S.s have been applied in a wide range of IoT applications, as illustrated in Table 1. This section will focus on the important roles of anomaly detection systems in industries, smart grids, and smart cities.

Industrial IoT is one of the beneficiaries of anomaly detection tools. Anomaly detection has been leveraged for industrial IoT applications such as power systems, health monitoring [28], heating ventilation and air conditioning system fault detection [29], production plant maintenance scheduling [30], and manufacturing quality control systems [31]. In [32], machine learning approaches such as linear regression have been applied to sensor readings of engine-based machines to learn deviations from normal system behaviours. The study demonstrated that anomaly detection plays a significant role in preventive maintenance by detecting machine failures and inefficiencies. In another study, autoencoder (A.E.)-based outlier detection was investigated in audio data using reconstruction error [33]. The study showed that early detection of anomalies could be used as responsive maintenance for machine failures, thereby reducing downtime. Furthermore, water facilities used IoT anomaly detection [34] to monitor and identify certain chemical concentration levels as a reactive alerting mechanism. These studies show that IoT anomaly detection provides mechanisms of improving efficiency and system up-time for industry machines by monitoring machine health.

The power sector including existing smart grids has also attracted anomaly detection systems to identify power faults and outages. The study in [35] utilised statistical methods to develop an anomaly detection framework using smart meter data. The authors argue that hierarchical network data can be used to model anomaly detection for power systems. The other study [36] employed high-frequency signals to detect anomalies in power network faults. The article concludes that local anomaly detection depends more on network size than topology. In [37], big data analysis schemes were explored to detect and localise failures and faults in power systems. The study showed that the compensation theorem in circuit theory could be applied to event detection in power networks. Physical attacks on smart grids such as energy theft can also be detected by using anomaly detection systems, as shown in [38]. It is compelling that anomaly detection plays a paramount role in detecting failures and faults in power systems, enhancing system reliability and efficiency.

Abnormality detection can be used for smart city facilities such as roads and buildings. Road surface anomalies were studied in [39]. It has been indicated that damage to private vehicles can be reduced if the road surface is monitored for anomalies so that timely measures such as maintenance are taken before road incidents. In the study undertaken in [40], pollution monitoring and controlling were modelled as an anomaly to enable policymaker decisions in health, traffic, and environment. Similarly, assisted living can also benefit from IoT-based anomaly detection as deviations from normal alert caregivers as studied in [41]. Thus, it can be summed up that abnormal situations in smart cities and buildings can be detected using anomaly detection systems, and these can be provided to policymakers for decision-making purposes.

## 3. Challenges in IoT Anomaly Detection Using Machine Learning

The development of anomaly detection schemes in the IoT environment is challenging due to several factors such as (1) scarcity of IoT resources; (2) profiling normal behaviours; (3) the dimensionality of data; (4) context information; and (5) the lack of resilient machine learning models [15]. These factors will be explained in this section.

### 3.1. Scarcity of IoT Resources

The leverage of device-level IoT anomaly detection can be hindered by the constraints in storage, processing, communication, and power resources. To compensate for this, the cloud can be adopted as a data collection, storage, and processing platform. However, the remoteness of the cloud can introduce high latency due to resource scheduling and round trip time. This delay may not be acceptable for real-time requirements of IoT suspicious events [15]. It is also evident that the scale of traffic in the IoT may degrade the detection performance of the anomaly detection system if it exceeds the capacity of the devices. A better solution is to offload certain storage and computations from devices to edge nodes or to send aggregated data to the cloud. Sliding window techniques can also offer reduced storage benefits by withholding only certain data points, though the anomaly detection system may require patterns/trends [26].

### 3.2. Profiling Normal Behaviours

The success of an anomaly detection system depends on gathering sufficient data about normal behaviours; however, defining normal activities is challenging. Due to their rare occurrence, anomalous behaviours might be collected within normal behaviours. There is a lack of datasets representing both IoT normal and abnormal data, making supervised learning impractical, specifically for massively deployed IoT devices. This drives the need to model IoT anomaly detection systems in unsupervised or semi-supervised schemes, where data deviating from those collected in normal operations are taken as anomalous [3].

### 3.3. Dimensionality of Data

IoT data can be univariate as key-value $x_{t}$ or multivariate as temporally correlated univariate $x_{t}=\left[x_{t}{}^{1},\ldots ,x_{t}{}^{n}\right]$. The IoT anomaly detection using univariate series compares current data against historical time series. In contrast, multivariate-based detection provides historical stream relationships and relationships among attributes at a given time. Thus, choosing a specific anomaly detection mechanism in IoT applications depends on data dimensionality due to associated overheads in processing [3,29]. Furthermore, multivariate data introduces the complexity of processing for models, which needs dimension reduction techniques using principal components analysis (P.C.A.) and A.E.s. On the other hand, univariate data may not represent finding patterns and correlations that enhance machine learning performance.

### 3.4. Context Information

The distributed nature of IoT devices caters to context information for anomaly detection. However, the challenge is to capture the temporal input at a time $t_{1}$ is related to input at a time $t_{n}$ and spatial contexts in large IoT deployments where some IoT devices are mobile in their operations. This means that introducing context enriches anomaly detection systems, but increases complexity if the right context is not captured [3].

### 3.5. Lack of Machine Learning Models Resiliency against Adversarial Attacks

The lack of a low false-positive rate of existing machine learning models and the vulnerability to adversarial attacks during training and detection call for both accurate algorithms and resilient models. On the other hand, the massive deployment of IoT devices could be leveraged for collective anomaly detection as most of the devices in the network exhibit similar characteristics. This large number of devices helps to utilise the power of cooperation against cyber-attacks such as malware [42]. Model poisoning and evasion can decrease the utility of machine learning models as adversaries can introduce fake data to train or tamper the model.

## 4. Machine Learning Techniques for Detecting Anomalies in the IoT

Several aspects of IoT anomaly detection using machine learning must be considered. Learning algorithm methods can be categorised into three groups: supervised, unsupervised, and semi-supervised. The technique to train the learning algorithms across many decentralised IoT devices is known as federated learning. In addition, anomaly detection can be seen in terms of extant data dimension, leading to univariate-and multivariate-based approaches. In the rest of this section, we will present the anomaly detection schemes based on (1) machine learning algorithms; (2) federated learning; and (3) data sources and dimensions.

### 4.1. Detection Schemes Based on Machine Learning Algorithms

Supervised algorithms, known as discriminative algorithms, are classification-based learning through labelled instances. These algorithms consist of classification algorithms such as the K-nearest neighbour (K.N.N.), support vector machine (SVM), Bayesian network, and neural network (N.N.) [43,44]. K.N.N. is one of the distance-based algorithms of anomaly detection where the distances of anomalous points from the majority of the dataset are greater than a specific threshold. Calculating the distances is computationally complex; it seems impossible to provide on-device anomaly detection using this algorithm. On the other hand, SVM provides a hyperplane that divides data points for classification. As in the case of K.N.N., it is so resource-intensive that the applicability to IoT anomaly detection is impractical. As the Bayesian network may not require the prior knowledge of neighbour nodes for anomaly detection, it can be adopted for resource-constrained devices through low accuracy. Finally, N.N. algorithms have been extensively used to train on normal data so that anomalous data can be detected as the deviation from normal. The resource requirements of N.N. algorithms make it challenging to adapt to the IoT environment. Hence, supervised algorithms are the least applicable for IoT anomaly detection systems for their labelled dataset requirements and extensive resource requirements.

Commonly known as generative algorithms, unsupervised algorithms use unlabelled data to learn hierarchical features. Clustering-based algorithms such as K-means and density-based spatial clustering of applications with noise (D.B.S.C.A.N.) are unsupervised techniques that apply similarity and density attributes to classify data points into clusters [43,44]. Abnormal points are small data points significantly far from the dense area, while normal points are either close to or within the clusters. Usually, clustering algorithms are used with classification algorithms to enhance anomaly detection accuracy. Because of resource usage, most of the clustering algorithms cannot be directly applied to IoT devices for anomaly detection. Another unsupervised learning technique involves dimension-reduction approaches such as P.C.A. and A.E. to remove noise and redundancy from data to reduce the dimension of original data [44,45]. P.C.A. has been extensively applied to anomaly detection, but it fails in the dynamic IoT environment. A.E. has produced promising results in IoT anomaly detection in reducing data sizes and in reconstructing errors to identify anomalous points. However, these techniques have been used extensively as a part of feature extraction for classification algorithms. The dimensionality reduction algorithms in unsupervised learning can be adapted to IoT anomaly detection. Semi-supervised algorithms combine discriminative and generative algorithms by providing normal data instances so that deviation from normal behaviour is seen as abnormal behaviour. Hence, anomaly detection in IoT is geared toward unsupervised or semi-supervised algorithms where normal system profiling is utilised as a baseline environment [46].

Table 2 shows the state-of-the-art machine learning algorithms according to three anomaly types.

### 4.2. Training Detection Schemes Based on Federated Learning Algorithms

Federated learning, also known as collaborative learning, allows IoT devices to train machine learning models locally and send the trained models, not the local data, to the server for aggregation [47,48]. This training method is different from the standard machine learning training approaches that require centralising the training data in one place such as a server or data centre.

The federating learning method consists of four main steps. First, the server initialises a global machine learning model for anomaly detection and selects a subset of IoT devices to send the initialised model. Second, each selected IoT device will train the model by using its local data, then send the trained model back to the server. Next, the server will aggregate received models to form the global model. Finally, the server will send the final model to all IoT devices to detect anomalies. Note that the server can repeat the tasks of selecting a sub-set of IoT devices, sending the global model, receiving the trained models, and aggregating the received models multiple times, as some devices may not be available at the time of federated computation or some may have dropped out during each round.

By using federated learning, data in the IoT system is decentralised, and data privacy is protected. The other advantages of federated learning include lower latency, less network load, less power consumption, and can be applied across multiple organisations. However, federated learning also suffers from some drawbacks such as inference attacks [49] and model poisoning [50].

### 4.3. Detection Mechanisms Based on Data Sources and Dimensions

Univariate IoT data consists of data representation from a single IoT device over time. In reality, anomaly detection systems utilise data from multiple IoT devices deployed in complex environments. These multivariate multi-sources feed richer contexts by providing noise-tolerant temporal and spatial information than a single source.

#### 4.3.1. Univariate Using Non-Regressive Scheme

In the non-regressive scheme, threshold-based mechanisms can be leveraged by setting low and high thresholds of observations on univariate stationary data to flag anomalies if a data point falls outside the boundary. More advanced mechanisms such as mean and variance thresholds produced over historical data can replace this min–max approach. Another similar approach is using a box plot to split data distribution into a range of small categories where new data points are compared against the boxes. These non-regressive approaches are ideal in saving resources such as processors and memories for IoT devices. However, being distributed techniques over univariate observations, the range-based schemes fail to detect contextual and collective anomalies due to the lack of the ability to capture temporal relationships [3].

N.N.s such as A.E.s, recurrent neural networks (R.N.N.), and long short-term memory (L.S.T.M.) can be used as non-regressive models to solve the problem of anomaly detection in the IoT ecosystem using univariate time series data. A.E. is used to reconstruct data symmetrically from the input to the output layer, and a high reconstruction error probably indicates abnormality [13]. A.E. can also be applied to resource-constrained IoT devices for conserving resources and battery power. On the other hand, R.N.N. provides memory in the network by affecting neurons from previous outputs through feedback loops. This enables the capture of temporal contexts over time. The vanishing gradient problem in R.N.N. makes it unsuitable for large IoT networks. L.S.T.M. can provide semi-supervised learning on normal time series data to identify anomaly sequences from reconstruction to solve this error problem. Hence, it seems that combining A.E. and L.S.T.M. can bring about resource-saving and accuracy requirements of the IoT anomaly detection tasks.

#### 4.3.2. Univariate Using Regressive Scheme

Predictive approaches, known as regressive schemes, enable identifying anomalies by comparing predicted value to actual value in time series data. Parametric models such as autoregressive moving average (A.R.M.A.) are popular techniques despite seasonality or mean shift problems in non-stationary datasets. However, these problems can be solved by using enhanced variants of A.R.M.A. such as autoregressive integrated moving average (A.R.I.M.A.) and seasonal A.R.M.A. As another approach to predictive IoT anomaly detection, NN-based predictive models such as M.L.P., R.N.N., L.S.T.M., and others can be applied to capture the dynamics of a time series on complex univariate data [46]. For instance, R.N.N., L.S.T.M., and G.R.U. models can represent the variability in time series data to predict the expected values for time sequences. Recently, attention-based models have been applied to IoT anomaly detection in complex long sequential data. Similar to the non-regressive scheme, sequential models can boost the accuracy of IoT anomaly detection if dimensional reduction algorithms can be used in feature extraction.

#### 4.3.3. Multivariate Using Regressive Scheme

As the additional variables increase data sizes, dimensionality reduction techniques such as P.C.A., A.E., and others can be employed to decrease overall data size. P.C.A. can capture the interdependence of variables for multivariate sources. It reduces the data size by decomposing multivariate data into a reduced set. The linearity and computational complexity of P.C.A. can limit its usage for IoT anomaly detection. A.E. works like P.C.A. and can discover anomalies in multivariate time series data using reconstruction error, the same way as in univariate cases. The promising aspect of A.E. is its low resource usage and its non-linear feature extraction. Similar to predictive and non-predictive models on univariate data, schemes using L.S.T.M., CNN, DBN, and others can also be applied to identifying anomalies in multi-source IoT systems. Specifically, CNN and L.S.T.M. algorithms can be preceded by A.E. for important feature extraction and resource savings. These deep learning schemes can learn spatio-temporal aspects of multivariate IoT data [12].

Clustering mechanisms are another approach to detect anomalies in multivariate data. In addition, graph networks can be used to learn models about variable or sequence relationships where the weakest weight between graph nodes is considered anomalous.

## 5. Analysis of Machine Learning for IoT Anomaly Detection

Anomaly detection systems have proven their capabilities of defending traditional networks by detecting suspicious behaviours. However, the standalone anomaly detection systems in classical systems do not fit the architecture of distributed IoT networks. In such systems, a single node compromise could damage the entire network. By collecting traffic from various spots, a collaborative anomaly detection framework plays a paramount role in thwarting cyber threats. However, the trust relationship and data sharing form two major challenges [42,51]. In this massive network, insider attacks can be a serious issue.

Furthermore, as most anomaly detection systems apply machine learning, nodes may not be willing to share normal profiles for training or performance optimisation due to privacy issues. The trust problem can be solved by implementing a central server that handles trust computation and data sharing. However, this approach could lead to a single point of failure and security, specifically for the large-scale deployment of IoT devices. Recently, blockchain has attracted much interest in financial sectors for its capability of forming trust among mistrusting entities using contracts and consensus. Blockchain could provide an opportunity to solve the problem of collaborative anomaly detection by providing trust management and a data-sharing platform. In the remainder of this section, we will focus on analysing (1) the collaborative architecture for IoT anomaly detection using blockchain; (2) datasets and algorithms for IoT anomaly detection; and (3) resource requirements of IoT anomaly detection.

### 5.1. Collaborative Architecture for IoT Anomaly Detection

Blockchain is a decentralised ledger that provides immutability, trustworthiness, authenticity, and accountability mechanisms for the maintained records based on majority consensus. Though it was originally applied to digital currency systems, blockchain can be applied in various fields. With the power of public-key cryptography, strong hash functions, and consensus algorithms, participating nodes in a blockchain can verify the formation of new blocks. A block typically consists of a group of records, timestamp, previous block hash, nonce, and a block’s hash. Thus, the change in a record or group of records will be reflected in the next block’s previous hash field, which makes it immune to adversarial change [42].

The powerful attributes of blockchain could provide a solid foundation for anomaly detection in distributed networks such as the IoT. IoT devices can collaboratively develop a global anomaly detection model from local models without adversarial attacks using blockchain architecture. As IoT needs mutual trust to share local models in a secure and tamper-proof way, consensus algorithms and decentralised blockchain storage make it challenging for malicious actors to manipulate the network. However, the successful Bitcoin consensus algorithms in financial areas such as proof-of-work require extensive storage and processing capabilities. Etherium has applied proof-of-stake where the participants’ stakes determine consensus. It uses smart contracts, and is less computationally intensive. Hyperledger Fabric is another customisable blockchain platform that applies smart contracts in distributed systems rather than cryptocurrencies. As it relies on central service to enable participants to endorse transactions, endorsing participants must agree on the value of a transaction to reflect changes in the local participant ledger. These three popular blockchain systems do not seem to solve resource-constrained IoT devices [51].

Blockchain-based security solutions have been discussed in a mix of traditional and IoT systems [52,53]. In these studies, a resource-rich device was connected to IoT devices, where the device acts as a proxy to connect IoT devices to the blockchain. A similar study was conducted in [54]. The main advantages of these approaches lie in resource savings, but they may also create a central point of failure. In [55], the author’s utilised smart contracts to integrate IoT devices into blockchain for communication integrity and authenticity through the resource requirement issues that may not make it practical. The most promising result has been achieved on distributed and collaborative IoT anomaly detection [51]. The study uses a self-attestation mechanism to establish a dynamic trusted model against which nodes compare to detect anomalous behaviour. The model is cooperatively updated by majority consensus before being distributed to peers.

### 5.2. Datasets and Algorithms for IoT Anomaly Detection

The lack of labelled realistic datasets has hampered anomaly detection research in the IoT. The existing data suffer from lacking realistic representation for IoT traffic patterns and lack capture of the full range of anomalies that may occur in the IoT. Class imbalance between normal traffic and anomalous patterns also manifests, which makes classification systems inefficient. Most IoT traffic can be represented as normal behaviour while it dynamically changes over time. As contextual information such as time, environment, and neighbour nodes profile rich information to improve anomaly detection in the IoT, it seems that multivariate data plays a significant role. The challenges associated with the absence of truly representative, realistic, and balanced datasets favour an anomaly detection scheme that profiles normal behaviours to detect anomalous points that deviate from the normal data [56]. Table 3 shows the common datasets that have been commonly used in some recent studies in this research area. As can be seen, most datasets are not specific to the IoT system; however, they are still suitable for training and evaluating anomaly-based I.D.S.s because they contain both normal and abnormal data.

The initial deployment of the IoT anomaly detection system lacks historical data that specify normal and anomalous points. This absence and the rare nature of anomalies challenge the usage of traditional machine learning schemes. Though several techniques of solving imbalanced data have been proposed, such methods cannot maintain the temporal context of anomalies. In addition, supervised algorithms capture only known anomalies while failing to detect novel attacks. Thus, unsupervised or semi-supervised approaches can be used to solve the limitations of supervised algorithms [54].

While several techniques have been used in IoT anomaly detection, most of the approaches have failed to satisfy the resource and power requirements of IoT devices [54]. Though there is no single best anomaly detection approach, deep learning techniques, specifically A.E. and CNN, have shown promising results in both delivering better resource-saving and accuracy, respectively [64]. While algorithms such as CNN and L.S.T.M. can boost detection accuracy, A.E. can be used to reduce the dimension of data and extract representative features by eliminating noise. Specifically, L.S.T.M. can be applied to dynamic and complex observations within time-series IoT data over a long sequence. Thus, it suggests that these techniques or combinations could be further explored to detect anomalies in the IoT ecosystem [65].

### 5.3. Resource Requirements of IoT Anomaly Detection

The resource-constrained nature of IoT devices prohibits the deployment of traditional host-based intrusion detection such as anti-malware and anti-virus. As traffic analysis consumes huge computational resources during anomaly detection, incremental approaches such as sliding windows can reduce the processing and storage requirements for IoT devices. It is also critical that the anomaly detection engine of the IoT system should operate in near real-time for reliable detection. This indicates that adaptive techniques help to improve the detection model over time without major retraining. However, offline training may be applied for initial deployment.

## 6. Conclusions

The IoT environment’s massive number, heterogeneity, and resource constraints have hindered cyber-attack prevention and detection capabilities. These characteristics attract monitoring IoT devices at the network level as on-device solutions are not feasible. To this end, anomaly detection is better positioned to protect the IoT network. To protect the system, anomaly detection is considered to be an important tool as it helps identify and alert abnormal activities in the system. Machine learning has been applied for anomaly detection systems in I.T. and IoT systems. However, the applications of anomaly detection systems using machine learning in I.T. systems have been better than the IoT ecosystem due to their resource capabilities and in-perimeter location. Nevertheless, the existing machine learning-based anomaly detection is vulnerable to adversarial attacks. This article has presented a comprehensive survey of anomaly detection using machine learning in the IoT system. The significance of anomaly detection, the challenges when developing anomaly detection systems, and the analysis of the used machine learning algorithms are provided. Finally, it has been recommended that blockchain technology can be applied to mitigate model corruption by adversaries where IoT devices can collaboratively produce a single model using blockchain consensus mechanisms. In the future, we plan to implement a blockchain-based anomaly detection system for protecting high-end IoT devices such as Raspberry Pi. The system can be built on a python-based machine learning platform such as TensorFlow and a blockchain platform such as Hyperledger Fabric, where Raspberry Pi devices act as distributed nodes.

## Figures and Tables

**Table 1:** Anomaly-Based I.D.S.s according to Anomaly Types and Applications.

		ANOMALY TYPES		
		Points	Contextual	Collective
**APPLICATIONS**	**Generic**	[6]	[7]	[8]
		[9]		[10]
				[11]
				[12]
				[13]
				[14]
				[15]
	**Flights**		[16]	
	**Industries**	[17]		
		[18]		
		[19]		
	**Health**		[20]	
	**Smart Cities**	[21]		
	**Smart Grids**		[22]	
	**Smart Home**	[23]		[24]
				[25]
				[26]
	**Unmanned Aerial Vehicles**		[27]	

**Table 2:** Learning Algorithms According to Anomaly Types and Machine Learning Schemes.

		ANOMALY TYPES		
		Points	Contextual	Collective
**MACHINE LEARNING SCHEMES**	**Supervised**	RF [21]	RL [16]	CNN [24]
		DL [17]	LSTM [22]	GNN [8]
				Multiple [10]
				AE-ANN [11]
				LSTM [12]
				AE-CNN [13]
				Ensemble [14]
	**Unsupervised**	AE-CNN [6]	Subspace [27]	AE [25]
		AE [18]		Self-learning [26]
	**Semi-Supervised**	TCN [23]	AE-LSTM [20]	DNN [15]
			DBN [7]	

**Table 3:** Common Datasets for Anomaly Detection in the IoT System (Adapted from [1]).

Dataset	Published Year	IoT Specific	Dimensions	Normal Instances	Abnormal Instances
N-BaIoT [57]	2018	Yes	115	555,932	6,545,967
CICIDS 2017 [58]	2017	No	80	2,273,097	557,646
AWID [59]	2015	No	155	530,785	44,858
UNSW-NB15 [60]	2015	No	49	2,218,761	321,283
NLS-KDD [61]	2009	No	43	77,054	71,463
Kyoto [62]	2006	No	24	50,033,015	43,043,255
KDD CUP 1999 [63]	1999	No	43	1,033,372	4,176,086
